# Adhesive Gold Foil

**Published:** 1870-07

**Authors:** Louis Jack

**Affiliations:** Philadelphia, Pa.


### ARTICLE VIII.

### *Adhesive Gold Foil.*

By Louis Jack, D.D.S., Philadelphia, Pa.

There appeared in the February number of the Dental Cosmos a very remarkable article. It is entitled "Adhesive Gold Foil: its Discovery, History, and Use," by Amos Westcott, M.D., D.D.S., etc. It will not be a dfficult task to show that, in the portion of the article relating to the discovery of adhesive foil, Dr. Westcott has made some serious mistakes in his claim, if he means that it has any connection with the practice of to-day; that he has made important omissions in the history of the subject; and on the use and application of it, he has said so little, that it is difficult to understand how it can be so important a discovery as he maintains it to be.

The character of Dr. Westcott's paper is such that a review of it might be made with considerable profit; but I prefer to confine myself to the endeavor to show that there has been no connection between what he claims to have done about the year 1840 and the important stride which has taken place in the practice of operative dentistry since 1855. It will easily be proven that there is a distinction between the observation of an uncertain and unreliable adhesiveness which gold foil was sometimes found to possess as much as thirty years ago, and that quality of excessive adhesiveness as now regularly furnished for our use, and as now appreciated by very many operators; between the blind observance of a property which, when marked, was then considered by nearly every one a difficulty and a serious impediment, and the intelligent estimation of this valuable property, which soon occasioned it to be turned into an important aid in filling carious teeth, once a clear definition of it had been made, accompanied by methods of using gold foil which were somewhat new.

I shall also make it plain to whom we are to credit the clear recognition of the extent of this property of pure gold, and his estimation of the importance of it, and to whose efforts the dental profession is indebted for having introduced and made public methods of operating which have, in the hands of so many operators, completely revolutionized the modes of filling teeth which were in general use as late as twenty years ago. It is not out of place that I should attempt to establish the claim of the one I believe to be entitled to whatever credit is connected with the introduction of extra adhesive foil into common use in our specialty. It happened that my associations at the time were such as to cause me to be familiar with the first tangible experiments in this direction, and that I was intimate with all the steps which led to its public announcement. I feel sure it will cause no surprise to the great majority of old practitioners, when I mentioned that the present methods of using adhesive gold foil will all be carried back for their starting-point to the published statements made by Dr. Robert Arthur about 1855. Before that time the generally practiced methods of introducing gold while filling teeth were such as to render inadmissible the use of foil possessing this property in more than a slight degree. Such a degree of adhesiveness as the bulk of dentists consider a great advantage to-day would then have been a constant source of complaint, on account of the methods of operating than in vogue. The manner of using gold then, as now, may be divided into two classes, viz., those where the layers of gold in being introduced were allowed or expected to slide upon each other, either in entering the cavity or in the after (consolidation, and those where the operator) consolidated the particles from the commencement, adding fresh pieces successively to that previously packed, until the carious cavity would be filled. This latter method was practiced by comparatively a very few persons, and only by such could any advantage be found in much adhesiveness. It could easily be shown that this latter mode did not reach a satisfactory realization until the character of adhesiveness was clearly made known, and until it was made a controllable quality by dentists; from this moment the latter method has constantly increased, and will continue to do so just so far as an intelligent knowledge of this quality fixes itself in the mind.

Any one who is not blinded by prejudice or wedded to familiar ways not easily parted from, must admit that from the time of the explanation by Dr. Arthur of the system he worked out for using adhesive gold, published from 1855 to 1857, and his expressed anticipations concerning the valuable results springing from the use of gold possessing this quality in a high degree, that a marked advance has been taking place in the quality of dental operations not previously attainable. It may be difficult for many to realize this, since it is hard for the mind to travel back into its old paths and regard them as any different from the present track. I could make clear, if space would permit, what changes have occured in the manner of preparing cavities and filling teeth consequent on this improvement, which changes have facilitated the performance of good fillings, as well as at the same time conducing to the better preservation of the teeth.

Now it so happened, as I have intimated, that I was cognizant of the facts connected with Dr. Arthur's realization of the great importance of the adhesiveness of gold; and these facts, as well as all the steps and methods connected with the subject, were published at that time; of these Dr. Westcott was aware, and yet he failed to take any notice of them in his article. It will be remembered that Dr. A. made haste, as an earnest of his appreciation of the valuable aid he found in his discovery of this property, and of the means to be used to regulate and control this adhesiveness, to publish a full statement of the whole subject. He exhibited to the whole profession how the adhesiveness could be increased and diminished at will; how and when to use it, and when not to accept it.

The circumstances connected with his claim will be found in the *News Letter* of that period, and in his treatise on the subject published by Jones & White in 1857. Of these facts Dr. Westcott must have been cognizant; his history of the subject is so far very deficient.

It may not be out of place here to enter into a relation of the mode of Dr. Arthur's independent discovery of this property. In the winter of 1855 I was associated with him in the practice of dentistry in Philadelphia. Dr. A. had been using "sponge gold" almost exclusively for a year previously; and (under his direction) I had been making experiments as to the relative density of "sponge gold" and gold foil fillings. While packing some foil which had become hard and unmanageable from handling and great age, he recommended me to aneal it, when he at once observed that it adhered in the same way in which the sponge gold did—in fact, that it required the same kind of manipulation. He at once commenced to use it in precisely the same manner as he had been using the sponge gold. Dr. A. quickly recognized the full importance of this, to him, newly-discovered property of gold, and he set to work earnestly, in his usual way, to investigate its character.

Mr. David Morgan, an enterprising and faithful manufacturer of gold foil, became at once interested in Dr. A.'s views; experimented with foil until he was soon able to produce with uniformity a gold of far greater adhesiveness than any which had been previously made. Before this time "sticky" gold was a somewhat accidental thing, the effort of the manufacturers being to overcome as far as they were able every considerable appearance of this quality. Other manufacturers also took the matter up, and now there is hardly any but have a far greater control of their production than they were previously capable of securing.

Quickly following this recognition came the necessity for a modification of the form of the cavity, of the mode of preparing the foil previously to introducing it, and also of the instruments to be used for the purpose. The common methods would not at all answer. This was a new step in dentistry, and the history of our speciality will, when examined, show that it has been one of the most marked advances which has yet been made in it. The introduction of sponge gold and the instruments necessary to use it certainly prepared the way for the effective use of adhesive gold; but considerable modification of the latter was necessary for the use of the newly-prepared foil. All the features of the modified manipulations were carefully worked out to a satisfactory conclusion, and the results anticipated were fully realized in practice.

When Dr. A found he had fallen on a new and valuable track, he lost no time in making known all he had done, in such a way as to be of service to the whole profession; and from the appearance of his two articles referred to, the general use of adhesive foil commenced, and has extended so widely as to be accepted at the present day by almost every good operator in the profession.

From the time referred to, the term "adhesive foil" began to be used, and nowhere will it be found at a date previous to this.

Dr. A. at that time was unaware of the adhesive qualities of gold. He went into an investigation of the matter as an entirely new thing, and from my knowledge of the circumstances I am confident this was the case. Not only was it new to Dr. A., but it was apparently entirely new to the profession; a strong presumptive evidence of this was the interest which the subject excited at the time, and the opposition which was made in many quarters as to the advantage to be derived from adhesiveness. It will be remembered that a considerable number denounced it as a dangerous innovation; others maintaining that it was a distinction without a difference. The first have had their answer in the modification of the methods of operating to secure the most perfect results, and the almost general acceptance of the property; the second may be enlightened by comparing what had been written on the subject before 1855 with what has been written since.

It will be remembered by those who know Dr. Arthur and his published statements, that some twenty years ago he was in the habit of using very heavy numbers of gold, ranging from 30 to 60. The process was one of working up solidly from a starting-point in the cavity, using very sharp points, somewhat as adhesive foil is used to-day; and yet Dr. Westcott, who, I am assured, was present at the meeting of the American Society when this method was first presented, did not take occasion to mention the application of what he now claims to have discovered long before that time. The use of heavy numbers of foil in this way was one of the steps which caused Dr. A. to use "sponge gold," and afterward again adhesive foil, around which circle he has completely traveled fully twice to my recollection.

I was a student of the Philadelphia College of Dental Surgery; attended the two first courses of lectures in this institution, and was present at nearly every lecture delivered by Dr. Townsend, to whom Dr. W. refers. He was a man we all know as having been a foremost operator of the time, and who was enthusiastically alert for every improvement, and free in his presentation of every method of any value in that department with which he was so closely identified, and in connection with which he is so widely known. And yet I never heard him give an intimation that he knew anything of "adhesive gold" until it was brought to his attention by Dr. Arthur. He could not have failed to be informed of the possession of any information of great value which was at all attainable. I remember nothing, either in his lectures or from his private conversation, that bore any more than the most remote relation to adhesive foil. I am sure he had no recognition of this quality beyond a certain small degree of adhesiveness about which there was then no uniformity. In proof of this he found Dr. A.'s methods and extra adhesiveness a difficulty he, in his established methods of operating, could not overcome. He is remembered to have stated that his brother, Charles Townsend, found the new method especially adapted to labial cavities. These facts are evidences of distinctiveness in both materials and methods.

I will call attention for a moment to the statement of Dr. Westcott, that he discovered this property accidentally about the year 1840. He states that in having a quantity of gold foil sent him by mail, the sheets of gold were found united in consequence of the leaves of paper having been removed, and that he at once accepted the hint that he might purposely take an advantage of this disposition of "sticky" gold, which has been to him of more or less service ever since. For his prescience in this I give him all credit, and the excellence of his operations all must admit who have become acquainted with them. Others as well as Dr. Westcott have recognized what he called the "stickiness" of gold, and it is unquestionably true that the productions of the better dentists of that time were due in a great measure to this property of the foil used; for, indeed, without adhesiveness in some degree, it were impossible at any time to make good fillings. But I fail to see in these facts any others than a common observation of a quality which, from the failure to fully appreciate it and work it up systematically, may be called a blind observation. Neither can I see any connection between what he claims he did then and the present methods of using adhesive gold foil. In this connection of the discovery adhesiveness, a noticeable feature in the matter is, that Dr. W. was one of the leading men in the dental profession at the period to which he refers. It was a time, it must be remembered, when dentistry was being delivered from the occult condition in which it had been for so long a time; this was the day when the first attempts to liberalize our specialty were made, and when its leading men were emulating each other in making and publishing improvements. The American Dental Society was then established, and the *American Journal of Dental Science* was started; in the former Dr. W. was a prominent and active member; and of the latter he was for a time an editor, and through a considerable period a frequent contributor to its pages. Now, what is remarkable is this, that, at a time when he was vying with others in making public any new views of his own, this great discovery will nowhere be found mentioned. It appears surprising that Dr. W. could have been satisfied with personal conversation simply concerning such an important discovery, and the more so when the zeal usually shown by him is considered. He could not then have had an inkling of the importance of a property he now claims the dis covery of, but for which he had not an intelligent appellation. I have no desire to question anything Dr. Westcott has said as to what he has done, or to detract anything from him which is his due. But when he attempts to write the history of adhesive gold foil as it has already developed itself, he has but little claim to confidence as a relator of past events unless he gives all the prominent facts connected with it.

It is also remarkable, that while Dr. W. claims the original discovery of adhesiveness, and declares it so great an improvement as wonld have warranted him in taking out a patent for it, that he does not avail himself of the advatages of this discovery, but uses gold in such away as to prelude the employment of foil possessing adhesiveness to any considerable degree. From my experience, if I were so limited in its use, I should endeavor to keep silent on the subject. So that it now appears that, after having discovered this valuable property thirty years ago, and having made use of it

"more or less ever since," Dr. W. has after his long silence, written nothing that is of the slightest value to any one who looks to him alone for instruction in the use of adhesive gold. Is it not dificult, therefore, to perceive what credit Dr. Westcott can expect to obtain for the mere fact of having accidentally discovered that gold had the property of welding in a cold state, although the discovery may have antedated that of any one else, unless he had brought it to the notice of the profession in such manner as to be serviceable to it? The use of adhesive gold has become so familiar to the dental profession at the present day, that the recollection of the disinterested spirit with which it was given to those who are now using it seems well-nigh forgotten. It affords me pleasure, as an act of justice, to recall attention to the real author of the method of using gold foil upon which most of the operators of to-day rely for their best results in their work.

In concluding, it may be stated, that it is a matter of little concern to whom we are indebted for improvements in our specialty, so long as they are well developed and clearly published; by so doing they are rendered beneficial to all. And as the world sees, he who does not act in this manner debars himself from any claim of originality, particularly when he happens to be a member of a liberal profession.

As no idea comes down into the world fully fledged, but may be found in many places at the same time, scattered as germs, so on y to that one who vivifies and develops the thought in an active and tangible way can any credit be given. When any new thing is cultivated in this manner, it is out of place and unfair for any of those who have had hold of a scattered germ to claim the maternity of the practically developed idea.

And beside, may I question whether it is not a hard thing that, while making a claim for improvements in any direction, any one should ignore those who have been investigating with some advantage and success in the same direction.
—*Dental Cosmos.*

